# Effects of dried chaya leaf meal inclusion in the diet on growth performance and blood profiles in Thai native chicken (*Pradu Hangdum*)

**DOI:** 10.5455/javar.2023.j651

**Published:** 2023-03-31

**Authors:** Morrakod Wongnhor, Worasin Malaithong, Duddoa Khonyoung

**Affiliations:** Animal Production Technology Division, Maejo University, Phrae Campus, Mae Sai, Thailand

**Keywords:** Blood profiles, dried chaya leaf meal, growth performance, Thai native chicken

## Abstract

**Objective:**

The objective of the present study was to evaluate the effects of using dried chaya leaf meal (DCLM) as a protein feed in a diet on growth performance, blood profiles, and carcass quality in a native Thai chicken.

**Materials and Methods:**

Eighty 14-day-old Pradu Hangdum chicks were divided into four groups with four replicates each: control (without DCLM), 10%, 20%, and 30% DCLM inclusion in mash feed. Growth performance was recorded weekly until 98 days of age. Blood profile, carcass quality, and visceral organ weight were measured at 98 days of age.

**Results:**

The 10%–30% DCLM inclusion in the diet did not affect feed intake or feed efficiency; however, the body weight gain of chicks decreased linearly with the increase in DCLM inclusion. The heterophils, eosinophils, and monocytes were linearly increased with the increasing DCLM levels in the groups. The serum blood chemistry did not differ among the groups, while the AST in 10% and 20% DCLM was lower than in the control. Increasing the level of DCLM in the chicken diet did not affect carcass quality.

**Conclusion:**

The DCLM can be used as a feed ingredient in Thai native chicken feed up to 20%.

## Introduction

The poultry industry is growing quickly because chicken meat is cheap and has high-quality protein. Pradu Hangdum is a native Thai chicken popular in rural areas because this breed is disease-tolerant. In addition, the meat is low in fat [1] and tastes good.

Feed is the most expensive part of raising chickens, especially protein-rich feed like soybean meal. Finding an alternative protein source that is available and cheap will benefit farmers. The Chaya plant (*Cnidoscolus chayamansa* Mc Vaugh) is an exciting plant that has been used for food by the Mayan people for many years [2]. This plant is a fast-growing tropical perennial shrub [3]. The chaya plant is also widely used as food in Thailand. Chaya leaves are rich in protein, and it has been reported that they contain a range of 27%–34.02% of crude protein on a dry matter basis [4,5]. The essential and non-essential amino acid profiles are balanced for humans and animals [6]. In addition, it was stated that chaya leaves contain polyphenols and alkaloids, which have antioxidant capacities and anti-inflammatory properties [7,8].

A blood profile is one way to monitor animal health. Also, enzyme activity, such as alkaline phosphatase (ALP), aspartate aminotransferase (AST), and alanine aminotransferase (ALT), is related to hepatocellular damage [9]. Onasanwo et al. [10] reported that feeding chaya to rats enhanced the hematological indices. Furthermore, some researchers reported that chaya leaf extract could reduce the damage to the liver and kidneys caused by toxic substances in rats [11,12]. Therefore, this study aimed to determine whether dried chaya leaf meal (DCLM) can be used as a source of a feedstuff with regard to growth performance, carcass quality, blood hematology, and serum biochemistry.

## Materials and Methods

### Ethical approval

For all feeding trials and data collection in the present study, the protocol was approved by animal care and used for science and technology research Maejo University Animal Care and Use Committee, Thailand (MACUC003P/2021).

### Chaya leaves preparation

Chaya leaves (*Cnidoscolus chayamansa*) were harvested from a home garden in the Rongkwang subdistrict, Phrae province, Thailand, and were sun-dried for 2 days. Afterward, the dried chaya leaves were finely milled and placed into plastic bags. The resulting DCLM was analyzed for its chemical composition, such as crude protein, fat, fiber, and ash, based on AOAC [13].

### Animal and feeding experiments

One hundred newly hatched chicks (Pradu Hangdum) were provided from the Rajamangala University of Technology Lanna hatchery and raised in heated brooders with a commercial starter basal diet. At 14 days of age, 80 birds were individually weighed and divided into 4 groups with 4 replicates of 5 chicks each: basal diet (control) and 10%, 20%, and 30% DCLM. Additionally, basal starter and finisher diets in mash form were fed from 14 to 42 and 43 to 98 days old, respectively (Table 1). The experimental diets of the four groups were formulated to provide equal amounts of crude protein and energy. The birds had free access to food and drinking water, and the chickens were housed in floor pens with rice hulls as litter under a daily lighting regimen of 22 h of light and an environmental room temperature. The growth parameters, such as feed intake (FI) and body weight (BW), were monitored weekly.

### Blood collection 

At the end of the experiment (98 days old), eight birds from each group (two birds per pen) underwent blood collection via wing vein. Two milliliters of blood were placed into a vacuum tube containing ethylenediaminetetraacetic potassium acid (BD Vacutainer^®^, Becton Drive, Franklin Lakes, USA) for the complete blood cells 3 ml of blood was placed in a vacuum tube and then centrifuged at 4,200 × gm for 5 min to collect serum. All blood samples and serum were stored below 4°C and analyzed within 24 h after collection. The white blood cells (WBC), such as heterophils, eosinophils, lymphocytes, and monocytes, were determined manually. The serum chemistries were determined by an automated blood chemistry analyzer (Automatic Clinical Chemistry Analyzer BA400).

### Relative carcass and internal organs weight

At 98 days of age, eight birds in each group from each replicate were randomly used to measure the carcass quality, length of the small intestine, and weight of internal organs. After decapitation, the entire visceral organs of the birds were detached. The internal organs, such as the liver, proventriculus, and gizzard, were weighed and calculated in relative BW. Moreover, the carcass quality of breasts, wings, drumsticks, and thighs was measured. 

**Table 1.** Feed formulations and nutrient composition of experimental diets (gm/kg).

Ingredients	Level of DCLM							
	14–42 days old				43–98 days old			
	0%	10%	20%	30%	0%	10%	20%	30%
Corn	610	573.4	481.3	387.4	503	600	560	489
Soybean meal (45% CP)	348.6	287.5	245	202.8	230	192	150.4	109
DCLM	0	100	200	300	0	100	200	300
Rice bran	0	0	0	0	233.5	73	25	5
Palm oil	0	16	52	89	0	10	39.9	73.1
Dicalcium phosphate	15.5	13	10.5	8	19.6	16.8	14.5	11.6
Calcium carbonate	18.8	0	0	0	7.6	0	0	0
Sodium chloride	2.5	2.5	2.5	2.5	2.5	2.5	2.5	2.5
DL-methionine	1.6	2.1	2.5	3	0.5	0.9	1.2	1.7
L-Lysine	0.5	3	3.7	4.8	0.8	2.5	4	5.6
Trace mineral-vitamin premix	2.5	2.5	2.5	2.5	2.5	2.5	2.5	2.5
Nutrient composition (Calculated)								
CP (%)	20.87	20.51	20.51	20.50	17.29	17.26	17.20	17.2
Metabolizable energy (kcal/kg of DM)	2,894	2,877	2,875	2,874	2,888	2,888	2,883	2,876

DCLM = DCLM, CP = Crude protein, DM = Dry matter.

### Statistical analyses

One-way analysis of variance was used to look at growth performance, blood profiles, and the relative weights of the carcass and the organs inside it. Statistically significant differences at the p < 0.05 level due to treatments were separated by least square difference using IBM Statistical Package for the Social Sciences Statistics for Windows (Version 26.0, Armonk, NY). In addition, the trend analysis was determined for linear and quadratic.

## Results

The composition of DCLM was 29.01% of crude protein, 12.74% of crude fiber, 8.76% of crude fat, and 8.92% of ash. 

### Growth performance

Compared with the control, FI and feed conversion ratio (FCR) in chicken fed in the DCLM inclusion groups were not significantly different (p > 0.05) (Table 2), while the BWG of chicken feed in the DCLM inclusion groups decreased linearly with the increasing levels of DCLM. The mortality rate of the 10% and 20% DCLM inclusions was zero, whereas the control and 30% DCLM had one bird dead each. 

### Blood hematology and serum chemistry

The blood hematology and serum biochemistry are shown in Table 3; the packed cell volume (PCV), red blood cell (RBC), hemoglobin, and WBC of chicken-fed DCLM were not different compared with the control (p > 0.05), whilst heterophils and monocytes linearly increased with increasing the DCLM inclusion in the diet. Although the DCLM inclusion in the diet did not significantly affect most of the serum chemistry parameters among the groups, the AST of chicken fed the 10% DCLM inclusion diet was the lowest (Table 4).

### Carcass quality, internal organ weight, and small intestinal length

The percentage of de-feathered carcasses and the relative weights of breasts, wings, drumsticks, and thighs, as well as abdominal fat, did not differ among the groups (Table 5). The liver and gallbladder weight of the DCLM inclusion in the diet group was higher than the control, while the proventriculus weight of the 20% and 30% DCLM inclusion groups was lower than that of the control. The length of the duodenum and jejunum of the 30% DCLM inclusion group was the shortest among all groups. 

## Discussion

The present DCLM has similar crude protein content compared with other reports that the protein content in chaya leaves ranged from 27% to 34.02% [4,5]. Their leaves have a higher protein content than those of other leafy plants, such as cassava leaves or Leucaena. It has been reported that chaya leaf meal (CLM) can be used as a feed ingredient up to 20% in blue shrimp [14]. An increase in BW gain (BWG) was found in the rats fed 5%, and 10% of CLM-supplemented diets [10]. The use of 15%–25% CLM inclusion in commercial diets showed similar BWG and FI compared with the chickens fed in the CLM-free group, but feed efficiency decreased in the CLM groups [15]. In the present study, using DCLM inclusion in diet also did not affect FI or FCR; however, the chickens in the high-level DCLM inclusion group linearly decreased in BWG, especially at the 30% DCLM level, which was lower than the control, whereas the 10% and 20% levels did not differ from the control group. The decrease in BWG may result from the higher fiber levels in the DCLM diet, as the crude fiber levels in the 0%, 10%, 20%, and 30% DCLM diets were 3.76%, 4.47%, 5.20%, and 5.95%, respectively.

Hematology is an application that can be used to diagnose animal health. In the present study, most of the hematology indices of the chickens fed a DCLM inclusion diet were not different when compared with the control. In contrast, the WBC count of chickens fed DCLM demonstrated a tendency to increase linearly (p = 0.07) along with the increasing amount of DCLM. In addition, heterophils, eosinophils, and monocytes were linearly increased in the birds fed DCLM inclusion diets compared with the control. Chaya leaves can modify the blood parameters in Wistar rats [10], and chaya leaf extract was shown to improve blood components in Wistar rats with induced diabetes [16].

**Table 2.** Effect of DCLM inclusion in the diet on growth performance in Thai native chicken (*n* = 4).

	Control	Level of DCLM			SEM	Contrast
		10%	20%	30%		
FI (gm)	4,521.91	4,368.70	4,177.39	4,095.54	98.71	NS
BWG (gm)	1,281.21^a^	1,201.92^ab^	1,185.94^ab^	1,110.49^b^	27.96	L
FCR	3.55	3.63	3.52	3.69	0.06	NS
Mortality (number)	1	0	0	1	-	-

SEM = Standard error of the mean, NS = Non-significant, L = Linear.

^a,b^ Means in the same row with different superscripts differ significantly (*p* < 0.05).

**Table 3.** Blood hematology of chicken-fed DCLM inclusion in diet (*n* = 8).

Parameter	Control	Level of DCLM			SEM	Contrast
		10%	20%	30%		
Hematology						
PCV (%)	26.75	26.37	26.25	27.50	0.37	NS
RBC (10^6^/ µl)	1.95	2.01	2.03	1.92	0.04	NS
Haemoglobin (gm/dl)	9.89	9.97	9.31	9.92	0.14	NS
WBC (10^3^/µl)	31.42	32.62	44.98	41.76	2.65	NS
Heterophil (%)	8.75	13.13	9.00	13.50	1.02	NS
Heterophil (10^3^/µl)	2.56^b^	4.36^ab^	4.19^ab^	5.43^a^	0.46	L
Eosinophil (%)	9.50	9.63	10.25	12.75	0.78	NS
Eosinophil (10^3^/µl)	2.89	2.87	4.94	5.22	0.46	L
Lymphocytes (%)	79.75^a^	75.38^ab^	78.12^ab^	70.25^b^	1.48	L
Lymphocytes (10^3^/µl)	25.36	24.76	34.69	29.71	2.11	NS
Monocytes (%)	2.00^b^	1.88^b^	2.63^ab^	3.50^a^	0.21	L
Monocytes (10^3^/µl)	0.61^b^	0.63^b^	1.16^a^	1.40^a^	0.11	L

PCV = Packed cell volume, RBC = Red blood cell, WBC = White blood cell, SEM = Standard error of the mean, NS = Non-significant, L = Linear.

^a,b^ Means in the same row with different superscripts differ significantly (*p* < 0.05).

**Table 4.** Serum chemistry of chicken-fed DCLM inclusion in diet (*n* = 8).

Parameter	Control	Level of DCLM			SEM	Contrast	Reference [19, 20]
		10%	20%	30%			
ALT (U/l)	3.00	2.57	2.87	2.25	0.24	NS	–
AST (U/l)	225.87^a^	182.71^b^	195.75^b^	199.75^ab^	5.22	Q	118–298
ALP (U/l)	1,003.71	1,083.83	1,088.14	1,276.14	80.99	NS	568–8,831
Albumin (gm/dl)	2.10	1.71	2.21	2.10	0.08	NS	1.3–2.8
Total protein (gm/dl)	4.28	3.87	4.40	4.24	0.09	NS	3.85–7

ALT = Alanine aminotransferase, AST = Aspartate aminotransferase, ALP = Alkaline Phosphatase, SEM = Standard error of the mean, NS = Non-significant, Q = Quadratic.

^a,b^ Means in the same row with different superscripts differ significantly (*p* < 0.05).

Serum enzyme activity, such as AST, ALT, and ALP, is a good indicator of cellular damage. Usually, these enzymes demonstrate greater activity in hepatocytes [17]. However, cell or liver injuries result in the leakage of enzymes into the bloodstream. In the present study, the AST value of chicken fed 10% and 20% DCLM inclusion in the diet was lower than that of the control group. The result was similar to that reported by Orji et al. [12], who reported that chaya leaf extract protected against lead-induced liver damage, resulting in decreased AST in the serum of rats. Our findings suggest that chaya leaves show a protective effect on hepatocyte cells.

In the present study, the relative weight of carcasses was not significantly different among the groups. Still, the relative visceral organ weight, such as the liver and gall bladder, of the 30% DCLM inclusion group, increased. On the other hand, the relative weight of the proventriculus was lower in the 30% DCLM inclusion group. The increased liver weight in this study may be related to the fiber in the experimental diet due to DCLM inclusion. Yokhana et al. [18] reported that an increase in the weight of the liver was found in chickens fed dietary fiber due to the increased activity of the liver [20].

In this study, we showed that adding a lot of DCLM to chickens’ diets didn’t help them grow faster. This may be because the high fiber content of DCLM is hard to digest and use, especially in young chicks. Before putting chaya leaves in chickens’ diets or doing experiments with chickens of different ages, more research needs to be done to look at their amino acid profiles.

**Table 5.** The relative weight of carcass, visceral organs, and relative length of the intestine of chicken fed DCLM inclusion in the diet at 98 days of age (*n* = 8).

Parameter	Control	Level of DCLM			SEM	Contrast
		10%	20%	30%		
%Defeathered carcass	82.86	80.67	83.13	80.33	0.55	NS
Carcass (gm/100 gm BW)						
Breast	13.49	13.10	15.20	13.21	0.39	NS
Wings	10.03	9.79	10.11	9.97	0.12	NS
Drum-sticks and thigh	24.34	24.65	25.01	24.10	0.25	NS
Abdominal fat	0.59	0.39	0.39	0.24	0.08	NS
Visceral organ (gm/100 gm BW)						
Liver with gallbladder	2.06^b^	2.38^a^	2.19^ab^	2.35^a^	0.05	Q
Gizzard	5.22	5.35	4.61	4.96	0.17	NS
Proventriculus	0.47^a^	0.48^a^	0.41^b^	0.38^b^	0.01	Q
Small intestinal length (cm/100 gm BW)						
Duodenum	1.83^ab^	1.92^a^	1.52^b^	1.95^a^	0.07	L, Q
Jejunum	3.41	3.20	2.96	3.80	0.12	NS
Ileum	3.18	3.53	3.03	3.63	0.11	NS

SEM = Standard error of the mean, NS = Non-significant, L = Linear Q = Quadratic.

^a,b^ Means in the same row with different superscripts differ significantly (*p* < 0.05).

## Conclusion

DCLM can be used as a feed ingredient for up to 20% of the diet, resulting in no adverse effects on growth performance, carcass quality, or cell integrity. It could be used to promote the immune system of Thai native chickens.

## List of abbreviations

ALT, alanine aminotransferase; ALP, Alkaline phosphatase; BWG, Body weight gain; BW, Body weight; DCLM, Dried chaya leaf meal; FCR, Feed conversion ratio; FI, Feed intake; WBC, White blood cell.
